# Characteristics of emotional gaze on threatening faces in children with autism spectrum disorders

**DOI:** 10.3389/fpsyt.2022.920821

**Published:** 2022-08-22

**Authors:** Yifan Zhang, Dandan Li, Tingting Yang, Chuanao Chen, Hong Li, Chunyan Zhu

**Affiliations:** ^1^The School of Mental Health and Psychological Sciences, Anhui Medical University, Hefei, China; ^2^Anhui Province Key Laboratory of Cognition and Neuropsychiatric Disorders, Hefei, China; ^3^Department of Neurology, First Affiliated Hospital, Anhui Medical University, Hefei, China; ^4^Anhui Province Hefei Kang Hua Rehabilitation Hospital, Hefei, China; ^5^Anhui Hospital Affiliated to the Pediatric Hospital of Fudan University, Hefei, China

**Keywords:** autism spectrum disorder, threatening faces, gaze characteristics, emotions, eye movement

## Abstract

Most evidence suggested that individuals with autism spectrum disorder (ASD) experienced gaze avoidance when looking at the eyes compared to typically developing (TD) individuals. Children with ASD magnified their fears when received threatening stimuli, resulting in a reduced duration of eye contact. Few studies have explored the gaze characteristics of children with ASD by dividing emotional faces into threatening and non-threatening pairs. In addition, although dynamic videos are more helpful in understanding the gaze characteristics of children with ASD, the experimental stimuli for some of the previous studies were still emotional pictures. We explored the viewing of dynamic threatening and non-threatening faces by children with ASD in different areas of interest (AOIs). In this study, 6–10 years old children with and without ASD viewed faces with threatening (fearful and angry) and non-threatening (sad and happy) expressions, respectively, with their eyes movements recorded. The results showed that when confronted with threatening faces, children with ASD, rather than TD, showed substantial eye avoidances, particularly non-specific avoidances in the fixation time on the mouths and significantly less time gazing at the mouths in any emotions, which was not observed for non-threatening faces. No correlations were found between the severity of symptoms and characteristics of gaze at the eyes and mouths in children with ASD. These results further enhance the understanding of the gaze characteristics of children with ASD on threatening and non-threatening faces and possibly provide additional evidence for their social interaction improvements.

## Introduction

As the prevalence of autism spectrum disorder (ASD) has been considerably increasing in the past few years, accumulating research is being conducted to investigate the symptoms and causes of ASD (1). The fifth edition of the Diagnostic and Statistical Manual of Mental Disorders (DSM-5) (2) indicates that the impaired social interaction and communication skills are core deficits in ASD. Currently, “the Intense World Theory” makes a point that individuals with ASD are overly sensitive and subsequently overreact to specific environmental stimuli, such as threatening faces, menacing behaviors, and potentially harmful interactions (3, 4). Neuroimaging studies have also demonstrated that when children with ASD are exposed to external stimuli, the amygdala is over-activated, which may lead to an amplified fear of threatening information, resulting in gaze avoidances (5). However, as yet, the processing features of threatening information have not been well studied.

The behavioral characteristics of autistic symptoms are mainly reflected in diminished orientation to faces and reduced eye contacts (6). In the past decade, researchers have generally categorized emotions as positive, neutral and negative, and have explored the gaze characteristics of children with ASD through eye movements (7–9). The majority of studies reported that children with ASD showed impaired recognitions of negative emotional faces (anger, fear, and sadness) (10–12). Yet further research into negative emotions has revealed inconsistent results. For example, Wang et al. found that children with ASD spent less time gazing at pictures of angry faces compared to typically developing (TD) children, while no reductions in orientation were observed for either sad or fearful faces (13). However, other researchers expressed a different view that individuals with ASD performed significantly worse when identified fearful faces, but rarely differed from children with TD when observed other emotions (14, 15). In fact, unlike sadness, fear and anger can be considered as emotions that contain threatening messages. Currently few studies have presented these two emotions as a class of experimental stimuli and it is still unclear whether children with ASD show reduced attention to faces when observe threatening emotional faces. Therefore, in this study, we further explored the gaze characteristics of children with ASD by classifying emotional faces as threatening (i.e., fear, anger) and non-threatening (i.e., sadness, happiness), respectively, building on previous research.

Emotional informations are generally conveyed through the core areas of the face (eyes and mouth) (16, 17), and more specifically, most of the social messages expressed by threatening emotions are conveyed by the eyes (18). One study found that when viewed pictures of angry faces, children with ASD showed an avoidance of the eyes compared to children with TD (19). Other researchers studying on images of angry and fearful faces inconsistently found that the ASD group did not show any eye avoidances and spent a similar duration of time gazing at the eyes as the TD group (20, 21). Most experimental materials used in previous studies were found to be static emotional face pictures which may not provide sufficiently detailed emotional expressions, probably accounting for the inconformity of the results. Contrary to static emotional faces, dynamic emotional faces can convey richer stimulus information, which is ecologically relevant and closer to daily life (22, 23). To our knowledge, few studies have chosen dynamic threat emotional faces as stimulus materials. In summary, in this study we sought to examine the gaze characteristics of children with ASD and TD in more detail by presenting dynamic threatening and non-threatening emotional faces in order to explore the differences of the patterns of areas when viewed emotional faces between the two groups of children.

Most “attention span” eye-tracking investigations focused on overall observation time, lacking a detailed analysis of time course. Yet a study found that individuals with ASD displayed active gaze avoidances at an early stage of perceiving emotional faces (24, 25). Another study gave the opposite outcome, namely that the ASD and TD groups performed similarly in the early stages of staring at emotional faces, whereas the gaze impairment in people with ASD occurred in the later stages of perception (26, 27). Consequently, we added a temporal-course analysis of the two groups of children gazing at different AOIs of threatening and non-threatening emotional faces, with the aim of examining when eyes avoidances occurred during the process of looking at diverse AOIs of different emotional faces in both groups and whether these eyes avoidances were moderated by different types of emotional faces or different AOIs.

There have been studies exploring the correlation between the severity of ASD and the gaze characteristics of eye movement. It was found that the less time they spent gazing at faces, the more severe their symptoms were in ASD in the view of the social scenes (28–30). Liu et al. also came to the same conclusion (31). Even so, relationship between the severity and the threatening faces in children with ASD were still unclear. In order to explore the role of symptom severity of children with ASD in focusing on different AOIs of different emotional faces, we further examined the correlation between the total Childhood Autism Rating Scale (CARS) scores and the gaze characteristics of the different AOIs of emotional faces.

In summary, the current study aimed to investigate firstly whether there were differences in gaze characteristics of different AOIs in dynamic threatening (i.e., fear and anger) and non-threatening emotional faces (i.e., sadness and happiness) between ASD and TD and secondly whether there were associations between the symptom scores of children with ASD and certain AOIs of each of the four emotional faces. This study proposed three hypothetical points: (1) In children with ASD, eyes avoidances appeared to be on the eyes of threatening emotion faces and mouth fixation time was lower for all four emotions compared to TD. (2) During the whole film, children with ASD avoid looking into the eyes of threatening emotional faces, and avoid gazing at the mouth of any emotional faces. (3) More severely symptomatic children with ASD were less likely to gaze at the eyes and mouth of threatening emotions.

## Materials and methods

### Participants

A total of 36 children with ASD aged between 5 and 10 years were recruited among children diagnosed with ASD by pediatric psychiatrists according to the diagnostic criteria for ASD in DSM-5 (2). All subjects can understand the instructional language and correct it by eye movements. Five children with ASD were excluded due to failed eye-tracking calibration or looking at the screen less than 50% of the time during the eye movement test. The TD group was collected from a public primary school in Hefei, Anhui Province. Thirty-six TD children aged between 7 and 10 years were recruited in total. Participants in both groups were matched for gender and age. All participants were right-handed, with normal vision and hearing and no history of other psychiatric or physical illnesses, and can understand the instructional language and correct it by eye movements.

Ultimately, a total of 31 people with ASD, ranging in age from 6 to10 years, and 36 TD controls participated and completed the study. For the quantification of data collection, parents of 28 children with ASD enrolled completed the CRAS. As shown in Table 1, the total scale score based on the CRAS was (31.79 ± 7.41). All participants voluntarily participated in the study. Before starting the experiment, we obtained written consent from all the children’s parents. This study was approved by the Ethics Committee of Anhui Medical University (approval number: 2019H024).

**TABLE 1:** The general information of ASD and TD participants (Mean ± SD).

	ASD (*n* = 31)	TD (*n* = 36)	*t/*x^2^	*p*
Gender (male/female)	29:2	30:6	0.375	0.540
Age (years)	6.94 ± 1.31	7.44 ± 0.84	0.348	0.730
CARS score	31.79 ± 7.41			

### Materials

#### Childhood autism rating scale

The CARS test widely used in the diagnostic assessment of children with ASD, consists of 15 items covering areas such as interpersonal relationships, emotional responses and visual reactions and is scored on a four-point scale from 1to 4, with a score of 1 indicating age-appropriate behavior and a score of 4 indicating severe abnormal behavior. A total score greater than or equal to 30 is judged to be autistic. A total score of 30–36 and 37–60 are, respectively considered as moderate and severe autism.

#### Eye-tracking paradigm

**FIGURE 1:**
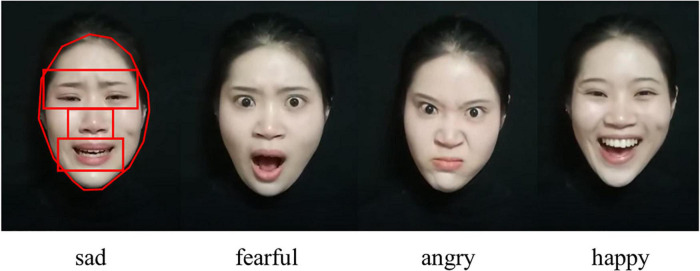
Four emotional films and AOIs.

The eye-tracking stimulus adapted from a previous study (30) was used in the present study of gaze characteristics of children with ASD and TD, on the basis of its application in our previous study of gaze characteristics of first-degree relatives of ASD and TD (32). The experimenters showed each participant four colorful films expressing four different emotions, namely, sad, fearful, angry and happy, respectively. The films all had the same scene—a girl dressed in black standing in front of a black background, with only the actress’ head and shoulders appearing in the films. Each film is silent, with the actress not speaking, but simply conveying different emotions dynamically and naturally to the camera (see Figure 1).

#### Apparatus

Eye movements were recorded by SMI’s iView X high-speed eye-tracking device (Senso Motoric Instruments, Germany). Experiment software was used to present films of facial emotions on a 30-inch monitor with a screen resolution of 1,680*960 pixels with 250 Hz. After the experiments were completed, we analyzed the eye-tracking data using BeGaze software (Gaze intelligence, Paris, France).

#### Procedure

The children were taken into a softly light and silent room. Their primary caregivers were asked to quietly stand beside them in order to give them a sense of security to complete the test. The children sat approximately 60 cm away from the monitor, facing it and gazing at it. Before starting the experiment, we performed the five-point calibration method of the SMI-red on each child. The calibration was accepted only when all five points of both eyes of the participants were captured by the SMI and the average error of each calibration point was less than 1° of visual angle. Children successfully calibrated will be included in the follow-up experimental study, and those not successfully calibrated will be excluded from the study.

At the beginning of the experiment, the command “Please watch the following movie carefully” appears in the display for 5,000 ms. Four films in a fixed order (sad, fearful, angry, and happy) were then played on the monitor and participants were asked to watch the films freely. Each film is presented for 20 s, and the whole paradigm is presented for about 80 s in total. At the end of the film, a “Thank you for watching” message appears on the monitor.

#### Data Analysis

After the experiment, we manually delineated the AOIs for each film using SMI-red eye-tracking software. We defined four AOIs on the actress’ face, namely, the full face, eyes, nose, and mouth (see Figure 1). The size of the AOI was consistent for all four films. We used BeGaze software to collect and store the eye-tracking data, which was finally exported and analyzed for each AOI.

Data of this study was statistically analyzed using SPSS 21.0 software. Demographic data and eye-tracking data were normally distributed, and a 2 (Group: ASD, TD) × 4 (Emotion: sad, fearful, angry, and happy) repeated measures analysis of variance (ANOVA) was used to compare the differences between ASD and TD groups in the characteristics of AOI gaze time for the four facial emotions. If there was an interaction between “group” and “emotion,” the *post hoc* test was continued. To further explore how the pattern of gaze on emotion changed over time for children with ASD vs. children with TD, the results of the experiment were analyzed for time course again. Each 20-s film of facial emotion was subdivided into twenty 1-s intervals. We used Pearson correlation analysis to examine the correlation between gaze duration and symptom severity.

## Results

### Fixation time on areas of interest

**FIGURE 2:**
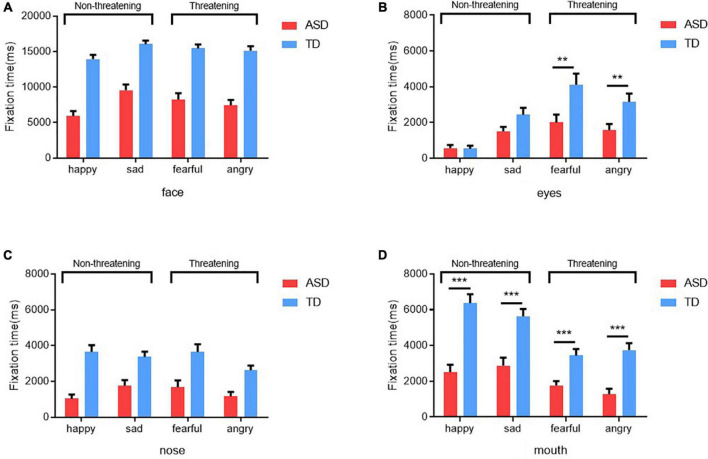
Gaze durations of the ASD and TD groups for the whole face **(A)**, eyes **(B)**, nose **(C)**, and mouth **(D)** in different emotional faces. **Denotes *p* < 0.01 and *** denotes *p* < 0.001.

In this study, a 2 (Group: ASD, TD) × 4 (Emotion: sad, fearful, angry and happy) repeated measures ANOVA was used to test for differences in the time spent gazing at different AOIs between the two groups. As shown in Figure 2, for eyes fixation time, there was a significant main effect of emotion [*F*_(3_, _256)_ = 21.91, *p* < 0.001, η^2^ = 0.51], a significant main effect of group [*F*_(1_, _64)_ = 7.41, *p* < 0.001, η^2^ = 0.10] and an interaction between emotion and group [*F*_(3_,_256)_ = 3.811, *p* < 0.05, η^2^ = 0.15]. *Post hoc* comparisons (after Bonferroni correction) showed that children with ASD spent significantly less time gazing at the eyes than those with TD when gazed at the emotional faces of fear and anger (fear: *p* = 0.009, anger: *p* = 0.009). However, no differences were found in the timing of gazing at the eyes of sad and happy emotional faces (see Figure 2B). The same approach can be used for the analysis of mouth fixation time. The results showed a significant emotion main effect [*F*_(3_, _256)_ = 21.93, *p* < 0.001, η^2^ = 0.51] and a significant group main effect [*F*_(1_, _64)_ = 37.97, *p* < 0.001, η^2^ = 0.37], with an interaction between emotion and group [*F*_(3_, _256)_ = 3.94, *p* < 0.05, η^2^ = 0.16]. *Post hoc* test analysis revealed that children with ASD had significantly shorter mouth gaze time for all emotions than those with TD (*p* < 0.001) (see Figure 2D). Otherwise, no differences were found between the two groups in terms of the duration of gaze on the full face and nose. Children with ASD and children with TD had similar gaze time for the full face and nose (see Figures 2A,D).

### Temporal-course analysis

**FIGURE 3:**
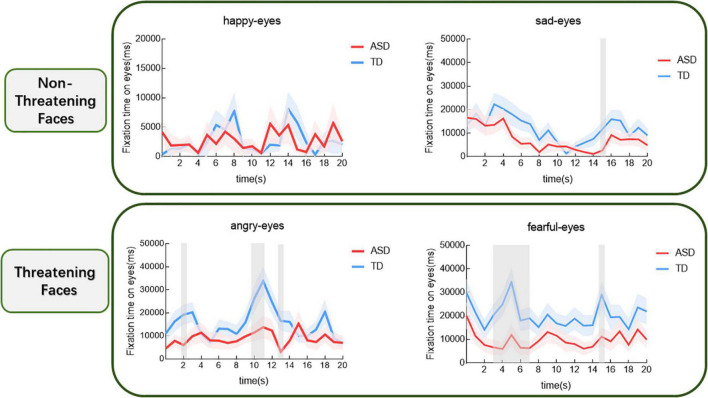
Proportional eye-looking time of the ASD and the TD groups for different expressions over time (shaded area indicates standard error). The gray shading indicates the cluster of time epochs when the group differences of face-looking time are significant.

Based on the results of the above analyses that the two groups differed in the fixation time of the eyes, we further investigated how the gaze characteristics of the different participants changed over time when watching different films. The results are shown in Figure 3. For the sad face, the group main effect was not significant (*t* = –1.94, *p* > 0.05). The comparison revealed that in the fifteenth second after the starting of the gazing children with ASD had a significantly shorter gaze to the eyes than those with TD (*p* < 0.05). For the fearful face, the group main effect was significant (*t* = –2.68, *p* < 0.01). In comparison, children with ASD spent significantly less time gazing at the eyes than those with TD, respectively, during the period from the third to the seventh second and in the fifteenth second after the starting of the gazing (*p* < 0.05). For angry face, the group main effect was significant (*t* = –2.68, *p* < 0.05). The comparison revealed that children with ASD spent significantly less time gazing at the eyes than those with TD, respectively, in the second second, during the period from the tenth to the eleventh second and in the thirteenth second (*p* < 0.05). However, for happy face, the group main effect was not significant and no significant differences were found between the two groups (*t* = 0.11, *p* > 0.05).

**FIGURE 4:**
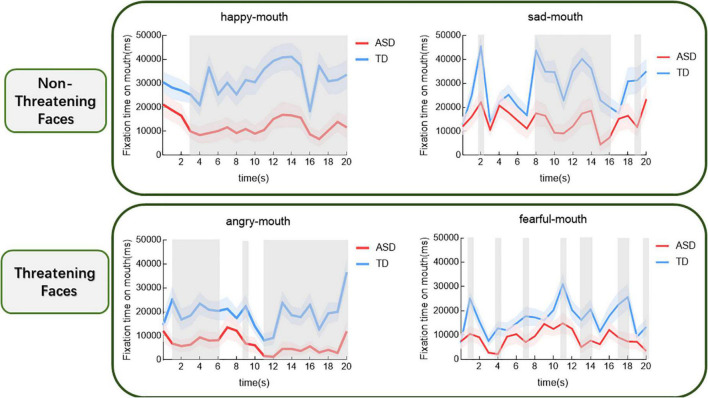
Proportional mouth-looking time of the ASD and the TD groups for different expressions over time (shaded area indicates standard errors). The gray shading indicates the cluster of time epochs when the group differences of face-looking time are significant.

In a way, we examined how the time spent gazing at different emotional mouths varied over time for both groups. The results are shown in Figure 4. For the sad face, the group main effect was significant (*t* = –4.53, *p* < 0.001). In contrast, children with ASD were found to gaze at the mouth for significantly shorter periods of time (*p* < 0.05) than those with TD, respectively, during the period from the eighth to the sixteenth second and in the nineteenth second. For the fearful face, the group main effect was significant (*t* = –3.68, *p* < 0.001). A significant difference in the duration of mouth gaze between the two groups was constantly found between the first and the twentieth second (*p* < 0.05). For the angry face, the group main effect was significant (*t* = –4.87, *p* < 0.001). Comparisons revealed that children with ASD spent significantly less time gazing at the eyes than those with TD between the first and sixth second, in the ninth second and between the eleventh and the twentieth second, respectively. For the happy face, a group main effect was significant (*t* = –5, 79, *p* < 0.001). The comparisons showed a significant difference of time gazing between the two groups during the period from the third to the twentieth second.

Correlations of eye-tracking variables and symptom traits

On all emotional face films, no correlations between eyes gaze time and total CARS scores were found for children with ASD (happy: *r* = 0.10, *p* = 0.601, sad: *r* = –0.05, *p* = 0.814, fearful: *r* = –0.17, *p* = 0.396, anger: *r* = –0.25, *p* = 0.196). We concluded that there was no association between symptom severity and emotional face-eye gaze characteristics in children with ASD. In addition, the outcome of the data analyses indicated that the time spent gazing at happy and fearful mouths by children with ASD was, respectively, negatively correlated with the total CARS score (happy: *r* = –0.42, *p* = 0.027, fearful: *r* = –0.39, *p* = 0.042). No significant correlations between symptom severity and face-eye gaze characteristics were found for either sad or angry faces (sad: *r* = –0.21, *p* = 0.290, angry: *r* = –0.23, *p* = 0.233) in children with ASD.

## Discussion

This research found eyes avoidances in children with ASD were more prominent when observed threatening emotional faces. Regardless of whether emotion was threatening or not, children with ASD spent less time viewing the mouths than those with TD. Results of the temporal-course analysis further indicated that children with ASD experienced persistent eyes avoidances of threatening emotions. For the fearful face, eyes avoidances in children with ASD appeared approximately in the third second after the presentation of the face and lasted up to the fifteenth second. For the angry face, children with ASD showed eyes avoidances approximately between the second second and thirteenth second. We rarely found group differences in happy and sad faces at any given period. Furthermore, the correlation analyses revealed no significant association between the severity of the clinical symptoms and the duration of time spent gazing, respectively, at the eyes and mouths in children with ASD, whatever the types of emotion the faces presented. However, the duration of gaze at the mouths of happy and angry faces was associated with symptom scores of children with ASD.

The results of our present study demonstrated that children with ASD spent less time looking at the fearful and angry eyes, but not happy and sad ones, when compared to those with TD. It also suggests that eyes avoidances in children with ASD may relate to threatening emotional stimuli. This specificity of social attention in children with ASD can also be explained by the “Intense World Theory” hypothesis that children with ASD are exposed to threatening sensory stimuli then triggering intense world phenomena, consequently resulting in avoidance of gazing at the relevant threatening information areas (eyes) in order to regulate internal homeostasis. Accordingly, children with ASD have a certain capacity for emotion recognition, however, they are more sensitive to threatening emotional faces, which induces them to show eyes avoidances to threatening stimuli rather than happy or sad stimuli. Researchers such as Kliemann also discovered that although individuals with ASD showed a low preference for fearful, happy and neutral face eyes, their attention to fearful eyes tend to be paid gradually but not quickly, which was found in neither happy nor neutral faces (33). It was also confirmed that children with ASD were more sensitive to the eyes of threatening emotional faces. Fearful and angry faces cause children with ASD to perceive threatening messages and feel discomfort, leading to eyes avoidances in order to alleviate this state (34, 35). Earlier studies have also pointed the possibility that ASD’s idiosyncratic pattern of attention to threatening faces may contribute to triggering their social avoidance behaviors, as well as impacts on the development of communicative interaction skills (36, 37). In clinical settings, eyes avoidance of emotional faces has become an important indicator for the early diagnosis of ASD (38).

In addition, our study also found that children with ASD exhibited a non-specific attention pattern of viewing the mouths, which was different from that of the eyes. For each of the four emotional faces, children with ASD spent fewer moments gazing at the mouths than those with TD. The children with ASD have shown significant avoidances of all emotional mouths. The same conclusion was reached in a previous study where children with ASD spent remarkably less time looking at the mouths in dynamic scenes than those with TD (39). A recent study similarly suggested that children with ASD paid significantly lower levels of attention to the mouths than those with TD control group (40). This may be due to the fact that the expression of emotions in the dynamic films makes the mouth move and change constantly and children with ASD have difficulty in recognizing emotions through the mouths. They are unable to accurately determine the type of emotion and thus experience avoidance across the whole range of emotions. Moreover, a correlation existed between the duration of time people with ASD spent watching the mouths and the level of speech (41, 42). Most people with ASD have a verbal impairment and therefore exhibit a non-specific fixation pattern on the mouths, and hence it is understandable that children with ASD spend significantly less time gazing at the mouths than those with TD.

When comparing the eye movements of the two groups of children during both threatening and non-threatening emotional faces scans based on temporal-course analysis, we discovered that children with ASD viewed the eyes in the threatening faces for shorter periods of time than those with TD, approximately from the second second to the fifteenth second after the face appeared, while no differences were found between the two groups in the duration of non-threatening faces eyes gazes. This was consistent with our assumed results that children with ASD showed persistent eyes avoidances of threatening emotional faces in the films. For the sad face, the difference in overall fixation time between the two groups was not significant and temporal-course analysis showed that children with ASD experienced eyes avoidances in the fifteenth second after the emergence of the face. Our analyses of the possible reason for this is that the actress’ expressiveness in the later stages of the sad emotional film was too strong and may have given a threatening message to the children with ASD, resulting in a brief eye avoidance that then disappeared. At the same time, the temporal-course analysis reconfirmed that children with ASD have specific fixation on the eyes and non-specific gaze pattern for the mouth. Throughout all the four emotional films, we found between-group differences in the duration of mouth fixation in both groups. Children with ASD spent shorter periods of time viewing the mouths than those with TD in any emotions. One researcher used static photos to find not only that developing children with ASD spent significantly less time gazing at the mouths compared to those with TD, but also that the fixation pattern was reversed during adolescence. Adolescents with ASD spent longer looking at the mouths than those with TD (43). That result indicated that the difference in mouth gaze patterns between ASD and TD changed with age, which, however, wasn’t reflected in our present study. Thus, combine the existing results, future examinations could explore the corresponding evidence for changes with increasing age in the different AOIs gaze characteristics of dynamic threatening and non-threatening emotion faces in individuals with ASD vs. TD.

Another innovation of this study was that we provided observations of differences in gaze patterns across AOIs of threatening and non-threatening emotional faces in the ASD group and examined their associations with symptom scores. We discovered that there was no correlation between the gazing time at the eyes and the total CARS score. Prior work found that a reduction in face fixation time could be a sensitive indicator of the degree of social functioning deficits in children with ASD (44). More severely symptomatic children with ASD had poorer facial recognition of anger, fear and sadness (45). Other researchers found a correlation between social attention to the mouth and ASD symptoms in the field of social communication (46). Our data did not find that the severity of symptoms in children with ASD was reflected by the time spent gazing at the eyes and mouth. The possible reason is supposedly that the clinical symptom score is one of the external phenotypes, whereas the gaze of the child with ASD is characterized by an internal phenotype. ASD also exhibits behavioral expressions such as stereotyped behaviors and limited interests. All of these appearance-based deficits have the potential to diminish their social motivation, making them spend less time gazing at faces (47, 48). At present, there are no definitive studies investigating the intrinsic link between the behavioral performances of children with ASD and their social attention and the reasons for it, which gives a novel direction for future studies on the correlation of symptoms in ASD.

There are also some limitations in the present study. Firstly, the participants in this study were mainly male and only included school-age children aged from 6 to 10 years in the end. In the future study, we can expand the sample size and age range of participants to explore differences in the gaze characteristics of ASD toward different emotional faces at different ages. Secondly, the experimental material for this study was selected only from female emotional faces and lacked neutral emotions stimuli. It is unclear whether it is easier to explore the differences in gazing at emotional faces for male emotional faces than female emotional faces in children with ASD and TD, and whether ASD still shows eyes avoidances on male threatening emotional faces, which need to be explored in the future study. Finally, we only used the CARS scale to score the symptoms of children with ASD. Future researches could add other scales, such as the Childhood Autism Behavior Scale (ABC), in order to explore the correlation between symptoms and eye movement characteristics of children with ASD in a much more detailed and precise way. These would also provide more insight into the clinical symptoms of children with ASD and offer more possibilities for the rehabilitation of ASD.

To conclude, we observed that children with ASD looked less at other’s eyes, especially the eyes conveying threatening emotional expressions. They spent less time fixating on the mouth of any emotional faces compared to children with TD. We consider that children with ASD have specific fixation on the eyes and non-specific fixation on the mouth, respectively, for threatening and non-threatening faces. Our trial filled the blanks in the study of the gaze characteristics of different AOIs when children with ASD were confronted with threatening faces and provided additional assistance in the early screening of ASD. However, we didn’t find strong evidence to support the association between higher scores on behavioral scales and gaze avoidances in ASD. Our data provided profitable insight into the distribution of gaze of children with ASD when viewing threatening emotional faces. Moreover, the scanning pattern for socially threatening facial expressions can also serve as a potential early marker of ASD. In the near future, it will be possible to combine our research findings with advanced information technology to integrate social attention with artificial intelligence in ASD, consequently offering more rehabilitation evidences for the treatment of social attention deficits in children with ASD.

## Data availability statement

The original contributions presented in this study are included in the article/supplementary material, further inquiries can be directed to the corresponding author/s.

## Ethics statement

The studies involving human participants were reviewed and approved by the Anhui Medical University. Written informed consent to participate in this study was provided by the participants’ legal guardian/next of kin.

## Author contributions

YZ, DL, HL, and CZ designed the study and wrote the article, which all authors have reviewed. TY and CC collected and analyzed the data. All authors approved the final version to be published and can certify that no other individuals not listed as authors have made substantial contributions to the manuscript.

## References

[1] Sun X Allison C Wei L Matthews FE Auyeung B Wu YY. Autism prevalence in China is comparable to Western prevalence. *Mol Autism.* (2019) 10:7. 10.1186/s13229-018-0246-0 30858963 PMC6394100

[2] American Psychiatric Association. *Diagnostic and Statistical Manual of Mental Disorders (DSM-5).* Washington, DC: American Psychiatric Pub (2013). books.9780890425596 10.1176/appi

[3] Markram H Rinaldi T Markram K. The Intense World Syndrome – an alternative hypothesis. *Front Neurosci.* (2007) 1:77–96. 10.3389/neuro.01.1.1.006.2007 18982120 PMC2518049

[4] Markram K Markram H. The intense world theory - a unifying theory of the neurobiology of autism. *Front Hum Neurosci.* (2010) 4:224. 10.3389/fnhum.2010.00224 21191475 PMC3010743

[5] Ghosn F Perea M Castelló J Vázquez MÁ Yáñez N Marcos I Attentional patterns to emotional faces versus scenes in children with autism spectrum disorders. *J Autism Dev Disord.* (2019) 49:1484–92. 10.1007/s10803-018-3847-8 30536217

[6] Franco F Itakura S Pomorska K Abramowski A Nikaido K Dimitriou D. Can children with autism read emotions from the eyes? The eyes test revisited. *Res Dev Disabil.* (2014) 35:1015–26. 10.1016/j.ridd.2014.01.037 24636022

[7] Reisinger DL Shaffer RC Horn PS Hong MP Pedapati EV Dominick KC. Atypical social attention and emotional face processing in autism spectrum disorder: insights from face scanning and pupillometry. *Front Integr Neurosci.* (2019) 13:76. 10.3389/fnint.2019.00076 32116580 PMC7026501

[8] Harrop C Jones D Zheng S Nowell SW Boyd BA Sasson N. Sex differences in social attention in autism spectrum disorder. *Autism Res.* (2018) 11:1264–75. 10.1002/aur.1997 30403327 PMC7468514

[9] Kleberg JL Nyström P Bölte S Falck-Ytter T. Sex differences in social attention in infants at risk for autism. *J Autism Dev Disord.* (2019) 49:1342–51. 10.1007/s10803-018-3799-z 30467821 PMC6450841

[10] Corden B Chilvers R Skuse D. Avoidance of emotionally arousing stimuli predicts social-perceptual impairment in Asperger’s syndrome. *Neuropsychologia.* (2008) 46:137–47. 10.1016/j.neuropsychologia.2007.08.005 17920642

[11] Bal E Harden E Lamb D Van Hecke AV Denver JW Porges SW. Emotion recognition in children with autism spectrum disorders: relations to eye gaze and autonomic state. *J Autism Dev Disord.* (2010) 40:358–70. 10.1007/s10803-009-0884-3 19885725

[12] Farran EK Branson A King BJ. Visual search for basic emotional expressions in autism; impaired processing of anger, fear and sadness, but a typical happy face advantage. *Res Autism Spectr Disord.* (2011) 5:455–62. 10.1016/j.rasd.2010.06.009

[13] Wang Q Lu L Zhang Q Fang F Zou X Yi L. Eye avoidance in young children with autism spectrum disorder is modulated by emotional facial expressions. *J Abnorm Psychol.* (2018) 127:722–32. 10.1037/abn0000372 30335441

[14] Guarnera M Hichy Z Cascio MI Carrubba S. Facial expressions and ability to recognize emotions from eyes or mouth in children. *Eur J Psychol.* (2015) 11:183–96. 10.5964/ejop.v11i2.890 27247651 PMC4873105

[15] Malaia E Cockerham D Rublein K. Visual integration of fear and anger emotional cues by children on the autism spectrum and neurotypical peers: an EEG study. *Neuropsychologia.* (2019) 126:138–46. 10.1016/j.neuropsychologia.2017.06.014 28633887

[16] Ekman P Friesen WV. Nonverbal leakage and clues to deception. *Psychiatry.* (1969) 32:88–106. 10.1080/00332747.1969.11023575 5779090

[17] Barrett LF Mesquita B Gendron M. Context in emotion perception. *Curr Direct Psychol Sci.* (2011) 20:286–90. 10.1177/0963721411422522

[18] van der Geest JN Kemner C Verbaten MN van Engeland H. Gaze behavior of children with pervasive developmental disorder toward human faces: a fixation time study. *J Child Psychol Psychiatry Allied Disciplines.* (2002) 43:669–78. 10.1111/1469-7610.00055 12120862

[19] Pérez-Edgar K Morales S LoBue V Taber-Thomas BC Allen EK Brown KM The impact of negative affect on attention patterns to threat across the first 2 years of life. *Dev Psychol.* (2017) 53:2219–32. 10.1037/dev0000408 29022722 PMC5705474

[20] Chawarska K Shic F. Looking but not seeing: atypical visual scanning and recognition of faces in 2 and 4-year-old children with autism spectrum disorder. *J Autism Dev Disord.* (2009) 39:1663–72. 10.1007/s10803-009-0803-7 19590943 PMC4878114

[21] Nakano T Tanaka K Endo Y Yamane Y Yamamoto T Nakano Y. Atypical gaze patterns in children and adults with autism spectrum disorders dissociated from developmental changes in gaze behaviour. *Proc Biol Sci.* (2010) 277:2935–43. 10.1098/rspb.2010.0587 20484237 PMC2982027

[22] Arsalidou M Morris D Taylor MJ. Converging evidence for the advantage of dynamic facial expressions. *Brain Topogr.* (2011) 24:149–63. 10.1007/s10548-011-0171-4 21350872

[23] Forni-Santos L Osorio FL. Influence of gender in the recognition of basic facial expressions: a critical literature review. *World J Psychiatry.* (2015) 5:342–51. 10.5498/wjp.v5.i3.342 26425447 PMC4582309

[24] Batty M Meaux E Wittemeyer K Roge B Taylor MJ. Early processing of emotional faces in children with autism: An event-related potential study. *J Exp Child Psychol.* (2011) 109:430–44. 10.1016/j.jecp.2011.02.001 21458825

[25] Wong TK Fung PC Chua SE McAlonan GM. Abnormal spatiotemporal processing of emotional facial expressions in childhood autism: dipole source analysis of event-related potentials. *Eur J Neurosci.* (2008) 28:407–16. 10.1111/j.1460-9568.2008.06328.x 18702712

[26] Senju A Johnson MH. Atypical eye contact in autism: models, mechanisms and development. *Neurosci Biobehav Rev.* (2009) 33:1204–14. 10.1016/j.neubiorev.2009.06.001 19538990

[27] Itier RJ Taylor MJ. Inversion and contrast polarity reversal affect both encoding and recognition processes of unfamiliar faces: a repetition study using ERPs. *Neuroimage.* (2002) 15:353–72. 10.1006/nimg.2001.0982 11798271

[28] Klin A Jones W Schultz R Volkmar F Cohen D. Visual fixation patterns during viewing of naturalistic social situations as predictors of social competence in individuals with autism. *Arch Gen Psychiatry.* (2002) 59:809–16. 10.1001/archpsyc.59.9.809 12215080

[29] de Wit TCJ Falck-Ytter T von Hofsten C. Young children with autism spectrum disorder look differently at positive versus negative emotional faces. *Res Autism Spectr Disord.* (2008) 2:651–9. 10.1016/j.rasd.2008.01.004

[30] Weiss EM Rominger C Hofer E Fink A Papousek I. Less differentiated facial responses to naturalistic films of another person’s emotional expressions in adolescents and adults with High-Functioning Autism Spectrum Disorder. *Prog Neuropsychopharmacol Biol Psychiatry.* (2019) 89:341–6. 10.1016/j.pnpbp.2018.10.007 30336172

[31] Liu Z Liu J Zhang Z Yu H Hu F. Facial emotion recognition and polymorphisms of dopaminergic pathway genes in children with ASD. *Behav Neurol.* (2020) 2020:6376842. 10.1155/2020/6376842 33204361 PMC7657692

[32] Yang T Li D Zhang Y Zhang L Li H Ji GJ. Eye avoidance of threatening facial expressions in parents of children with ASD. *Neuropsychiatr Dis Treat.* (2021) 17:1869–79. 10.2147/NDT.S300491 34140771 PMC8203098

[33] Kliemann D Dziobek I Hatri A Steimke R Heekeren HR. Atypical reflexive gaze patterns on emotional faces in autism spectrum disorders. *J Neurosci.* (2010) 30:12281–7. 10.1523/JNEUROSCI.0688-10.2010 20844124 PMC6633461

[34] Hutt C Ounsted C. The biological significance of gaze aversion with particular reference to the syndrome of infantile autism. *Behav sci.* (1966) 11:346–56. 10.1002/bs.3830110504 5970485

[35] Tanaka JW Sung A. The “eye avoidance”. hypothesis of autism face processing. *J Autism Dev Disord.* (2016) 46:1538–52. 10.1007/s10803-013-1976-7 24150885 PMC3997654

[36] Zalla T Sperduti M. The amygdala and the relevance detection theory of autism: an evolutionary perspective. *Front Hum Neurosci.* (2013) 7:894. 10.3389/fnhum.2013.00894 24416006 PMC3874476

[37] Garcia-Blanco A Lopez-Soler C Vento M Garcia-Blanco MC Gago B Perea M. Communication deficits and avoidance of angry faces in children with autism spectrum disorder. *Res Dev Disabil.* (2017) 62:218–26. 10.1016/j.ridd.2017.02.002 28214050

[38] Chawarska K Macari S Shic F. Context modulates attention to social scenes in toddlers with autism. *J Child Psychol Psychiatry.* (2012) 53:903–13. 10.1111/j.1469-7610.2012.02538.x 22428993 PMC3845814

[39] Gepner B Godde A Charrier A Carvalho N Tardif C. Reducing facial dynamics’ speed during speech enhances attention to mouth in children with autism spectrum disorder: an eye-tracking study. *Dev Psychopathol.* (2021) 33:1006–15. 10.1017/S0954579420000292 32378498

[40] Falck-Ytter T Fernell E Gillberg C von Hofsten C. Face scanning distinguishes social from communication impairments in autism. *Dev Sci.* (2010) 13:864–75. 10.1111/j.1467-7687.2009.00942.x 20977557

[41] Elsabbagh M Bedford R Senju A Charman T Pickles A Johnson MH. What you see is what you get: contextual modulation of face scanning in typical and atypical development. *Soc Cogn Affect Neurosci.* (2014) 9:538–43. 10.1093/scan/nst012 23386743 PMC3989131

[42] Fedor J Lynn A Foran W DiCicco-Bloom J Luna B O’Hearn K. Patterns of fixation during face recognition: differences in autism across age. *Autism.* (2018) 22:866–80. 10.1177/1362361317714989 28782371 PMC6599607

[43] Safra L Ioannou C Amsellem F Delorme R Chevallier C. Distinct effects of social motivation on face evaluations in adolescents with and without autism. *Sci Rep.* (2018) 8:10648. 10.1038/s41598-018-28514-7 30006527 PMC6045598

[44] Blair RJR Melanie C. Expression recognition and behavioural problems in early adolescence. *Cogn Dev.* (2000) 15:421–34. 10.1016/s0885-2014(01)00039-9

[45] Matson JL Nebel-Schwalm MS. Comorbid psychopathology with autism spectrum disorder in children: an overview. *Res Dev Disabil.* (2007) 28:341–52. 10.1016/j.ridd.2005.12.004 16765022

[46] Ketelaars MP In’t Velt A Mol A Swaab H Bodrij F van Rijn S. Social attention and autism symptoms in high functioning women with autism spectrum disorders. *Res Dev Disabil.* (2017) 64:78–86. 10.1016/j.ridd.2017.03.005 28376324

[47] Simonoff E Pickles A Charman T Chandler S Loucas T Baird G. Psychiatric disorders in children with autism spectrum disorders: prevalence, comorbidity, and associated factors in a population-derived sample. *J Am Acad Child Adolesc Psychiatry.* (2008) 47:921–9. 10.1097/CHI.0b013e318179964f 18645422

[48] Horstmann G Bauland A. Search asymmetries with real faces: testing the anger-superiority effect. *Emotion.* (2006) 6:193–207. 10.1037/1528-3542.6.2.193 16768552

